# Prevalence and predictors of Post-Acute COVID-19 Syndrome (PACS) after hospital discharge: A cohort study with 4 months median follow-up

**DOI:** 10.1371/journal.pone.0260568

**Published:** 2021-12-07

**Authors:** Imad M. Tleyjeh, Basema Saddik, Nourah AlSwaidan, Ahmed AlAnazi, Rakhee K. Ramakrishnan, Deema Alhazmi, Ahmad Aloufi, Fahad AlSumait, Elie Berbari, Rabih Halwani

**Affiliations:** 1 Infectious Diseases Section, Department of Medical Specialties, King Fahad Medical City, Riyadh, Saudi Arabia; 2 College of Medicine, Alfaisal University, Riyadh, Saudi Arabia; 3 Division of Infectious Diseases, Mayo Clinic College of Medicine and Science, Rochester, MN, United States of America; 4 Division of Epidemiology, Mayo Clinic College of Medicine and Science, Rochester, MN, United States of America; 5 College of Medicine, University of Sharjah, Sharjah, United Arab Emirates; 6 Sharjah Institute for Medical Research, University of Sharjah, Sharjah, United Arab Emirates; 7 Department of Medical Specialties, King Fahad Medical City, Riyadh, Saudi Arabia; 8 Department of Pediatrics, Prince Abdullah Ben Khaled Celiac Disease Research Chair, Faculty of Medicine, King Saud University, Riyadh, Saudi Arabia; Ohio State University Wexner Medical Center Department of Surgery, UNITED STATES

## Abstract

**Background:**

Post-acute COVID-19 syndrome (PACS) is an emerging healthcare burden. The risk factors associated with PACS remain largely unclear. The aim of this study was to evaluate the frequency of new or persistent symptoms in COVID-19 patients post hospital discharge and identify associated risk factors.

**Methods:**

Our prospective cohort comprised of PCR-confirmed COVID-19 patients admitted to King Fahad Medical City, Riyadh, Saudi Arabia between May and July 2020. The patients were interviewed through phone calls by trained physicians from 6 weeks up to 6 months post hospital discharge. Multivariate Cox proportional hazards and logistic regression models were used to examine for predictors associated with persistence of symptoms and non-return to baseline health.

**Results:**

222 COVID-19 patients responded to follow-up phone interviews after a median of 122 days post discharge. The majority of patients were men (77%) with mean age of 52.47 (± 13.95) years. 56.3% of patients complained of persistent symptoms; 66 (29.7%) experiencing them for >21 days and 64 (28.8%) reporting not having returned to their baseline health. Furthermore, 39 patients (17.6%) reported visiting an emergency room post discharge for COVID-19-related symptoms while 16 (7.2%) had required re-hospitalization. Shortness of breath (40.1%), cough (27.5%) and fatigue (29.7%) were the most frequently reported symptoms at follow-up. After multivariable adjustments, female gender, pre-existing hypertension and length of hospital stay were associated with an increased risk of new or persistent symptoms. Age, pre-existing lung disease and emergency room visits increased the likelihood of not fully recovering from acute COVID-19. Patients who were treated with interferon β-1b based triple antiviral therapy during hospital stay were less likely to experience new or persistent symptoms and more likely to return to their baseline health.

**Conclusions:**

COVID-19 survivors continued to suffer from dyspnea, cough and fatigue at 4 months post hospital discharge. Several risk factors could predict which patients are more likely to experience PACS and may benefit from individualized follow-up and rehabilitation programs.

## Introduction

More than one year into the Coronavirus disease 2019 (COVID-19) pandemic, several parts of the world are still experiencing high level of new cases and hospitalizations. To add to this burden, a sizeable subset of patients who have recovered from acute COVID-19 infection have reported lingering symptoms, leading to disability and impairment of their daily life activities. These patients are considered to suffer from “long” or “chronic” COVID-19 or a form of post-acute COVID-19 syndrome. Patients experiencing this syndrome have been coined COVID-19 long-haulers [1]. Severe acute respiratory syndrome coronavirus 2 (SARS-CoV-2) affects not just the respiratory system but multiple others including cardiovascular, brain, liver, kidneys, and gastrointestinal tract [2]. Despite recovery from the active viral phase, the persistence of atypical chronic symptoms, including extreme fatigue, shortness of breath, joint pains, brain fogs and mood swings, implies an underlying pathology that persists beyond the acute presentation of the disease [3–5]. We recently hypothesized plausible mechanisms underlying these long-term symptoms and described the multi-organ long-term manifestations of COVID-19 [6]. COVID-19 long-haulers thus, present with a disease portrait distinct from the typical acute COVID-19 disease displaying persistent symptoms that appear to be independent of disease severity. The full spectrum of these long-term health consequences is not yet fully understood.

There is no standardized definition of “long COVID” or “post-acute COVID-19 syndrome (PACS)”. The National Institute for Health and Care Excellence (NICE) defined it as a constellation of “signs and symptoms that develop during or after an infection consistent with COVID-19, continue for more than 12 weeks and are not explained by an alternative diagnosis” [7]. However, the French health authorities defined long COVID-19 as one with symptoms that persist beyond 4 weeks from the onset of acute illness [8]. Another group of investigators defined PACS as persistent symptoms and/or delayed or long-term complications of SARS-CoV-2 infection beyond 4 weeks from the onset of symptoms [1]. It is further divided into two categories: (1) subacute or ongoing symptomatic COVID-19, which includes symptoms and abnormalities present from 4–12 weeks beyond acute COVID-19; and (2) chronic or post-COVID-19 syndrome, which includes symptoms and abnormalities persisting or present beyond 12 weeks of the onset of acute COVID-19 and not attributable to alternative diagnoses [9, 10].

Symptoms have been reported to persist for as long as 9 months after onset [11]. In addition to the critically ill and hospitalized COVID-19 patients, outpatients with mild COVID-19 were observed to experience a delayed return to their baseline health [12]. To date, there are no published data about the prevalence of PACS in many parts of the world including the Middle East and North Africa (MENA) region. Therefore, we sought to describe the prevalence of PACS symptoms in a cohort of COVID-19 patients after hospital discharge and to examine for predictors of new or persistent symptoms and lack of return to baseline health status at follow-up.

## Materials and methods

### Study design, setting and participants

This cohort study was conducted at King Fahad Medical City (KFMC), Riyadh, Kingdom of Saudi Arabia. The study included all participants who were 18 years of age or older and were admitted to KFMC with Polymerase Chain Reaction (PCR)-confirmed COVID-19 infection during the months of May until July in 2020. We excluded patients who died, those who continued to be hospitalized at the time of follow-up, those who were unable to participate due to a mental illness, and those who failed to respond to phone calls after discharge.

### Data collection procedures

A structured questionnaire was used to extract clinical and demographic characteristics (including age, gender, nationality) from the patient’s hospital records. Data collected included presence of medical comorbidities, pre-defined symptoms at presentation and hospital course. Enrolled patients were subdivided into intensive care unit (ICU) or general ward admission, and disease severity (mild, moderate, severe, and critical) according to the WHO/ISARIC COVID-19 clinical characterization and patient’s utilization of supplemental oxygen need and hemodynamics during admission. In accordance with WHO guidelines, a seven-category ordinal scale was further used to characterize disease severity during hospital stay [13, 14]. Accordingly, the seven-category scale consisted of the following categories: 1, not hospitalized with resumption of normal activities; 2, not hospitalized, but unable to resume normal activities; 3, hospitalized, not requiring supplemental oxygen; 4, hospitalized, requiring supplemental oxygen; 5, hospitalized, requiring nasal high-flow oxygen therapy, noninvasive mechanical ventilation, or both; 6, hospitalized, requiring ECMO, invasive mechanical ventilation, or both; and 7, death. Treatment protocol was classified into antivirals available at KFMC at the time and included Favipiravir, convalescent plasma, and triple antiviral therapy (lopinavir/ritonavir + ribavirin + interferonβ-1b), immunomodulators which included corticosteroids and Tocilizumab, and supportive therapy. Chest roentgenogram at admission, clinical data, and laboratory reports during acute COVID-19 infection were also retrieved from the participants’ electronic medical records.

Participants were contacted through phone calls by trained physicians from 6 weeks to 6 months after hospital discharge to collect information on unresolved symptoms at follow-up, and duration for resolution of symptoms. In order to ensure that data were collected in a standardized manner, a structured interview process was used to collect information from patients. General interviewer training which covered the basics of interviewing skills, probing, and how to avoid refusals was conducted, in addition to research-specific training including a complete review of the data collection instrument, the time that should be taken to conduct the interview, and how to address questions from patients. All interviewers were trained to follow a set script and protocol of questions to ensure the consistency and structure of questions (S1 and S2 Questionnaires). A pilot study followed by a debriefing was conducted prior to data collection to identify and minimize potential bias and inconsistency between interviewers; as well as to ensure clarity, consistency, face and content validity of the survey questions and response items. Additionally, periodic meetings with the research team were held to ensure no divergence among the team and data collection had occurred. On average, the survey took approximately 10 minutes to complete. The interviews were conducted in Arabic or English based on patients’ language fluency. Two patients could only speak other languages and were therefore excluded. The aim of the phone call and research objectives were explained to the patients and verbal consent for participation was sought. During the phone interview, patients were asked for follow-up on their COVID-19 residual symptoms that lasted <8, 8–14, 15–21 and >21 days post discharge. Patients were also asked to report whether they felt they had completely returned to their pre-COVID state. “Non return to baseline” as an outcome was a subjective feeling by the patient. This outcome was not validated further. Like all symptoms reported by the patient, it is an assessment of how the patient felt overall at follow- up.

### Ethics

The study protocol was approved by the Institutional Review Board (IRB) at KFMC (IRB No. 20–557). All participants gave their verbal informed consent to participate in the telephone interviews.

### Statistical analyses

Demographic, clinical characteristics, and symptoms at presentation and follow-up were presented in means (and standard deviations [SD]) and medians (and interquartile ranges [IQR]) for continuous variables, and expressed as absolute values and percentages for categorical variables. For the comparison of symptoms at follow-up with demographic and clinical characteristics, the Pearson’s Chi-Square (χ2), or Fisher’s exact test were used for categorical variables and the Mann-Whitney-Wilcoxon and Kruskal Wallis tests were used for ranked data, as appropriate. Data were tested for normality visually using histograms and Q-Q plots, and statistically using Kolmogorov-Smirnov test. Homogeneity of variance was tested using Levene’s test.

A multivariate Cox proportional hazards model was constructed to identify factors associated with persistence of symptoms at follow-up with time-dependent days since discharge. Only statistically significant variables in the univariate analysis were incorporated into the multivariate Cox regression model. To assess the proportional hazards assumption, an extended COX regression model with time covariate interactions tested the interaction between time and covariates in the model. Predictor by time interactions for all predictors were non-significant (*p>0*.*05)* and did not violate the assumption of proportional hazards. The estimates of risk of predictor variables and persistence of symptoms at follow-up (event) were reported as Hazards ratio (HR) with a 95% confidence interval (CI).

To identify potential risk factors for non-return to baseline health status, a multivariable logistic regression analysis was performed. Variables with a p<0.05 in the univariate analysis were included in the model. Diagnostic measures of collinearity (tolerance and variance inflation factor (VIF) showed that multicollinearity was not a problem and assumptions of collinearity were met. Variable tolerances ranged between (0.71–0.96) and VIFs between (1.09–1.40). The automatic selection of predictors in the model was performed by a stepwise backward method with an entry threshold of 0.05 and an exit threshold of 0.1. The adequacy of the model was verified by the Hosmer and Lemeshow test, and its specificity by Link Test. The estimates of the strengths of associations for the multivariable logistic regression analyses were reported as odds ratio (OR) with a 95% confidence interval (CI).

The percentage of missing values across variables in the dataset varied between 0 and 7%. Multiple imputation was used to create and analyze 5 imputed datasets to account for missing data. All statistical analyses were performed using IBM SPSS version 26, (Chicago, IL) software. A two-tailed p < 0.05 was considered statistically significant.

## Results

**Fig 1:**
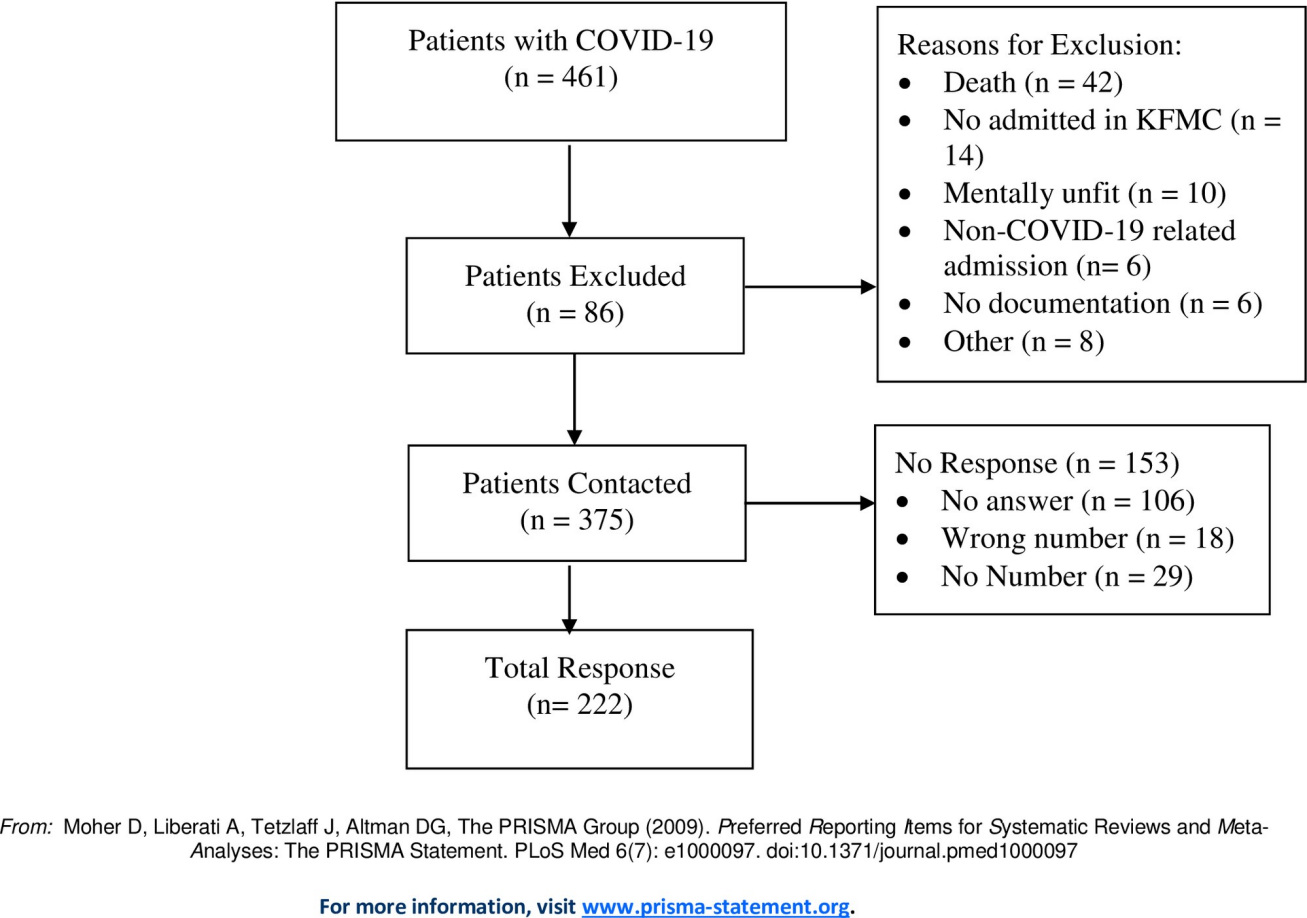
Flow diagram of COVID-19 patients hospitalized at KFMC between May and July, 2020.

A total of 461 patients were hospitalized at KFMC with PCR-confirmed SARS-CoV-2 infection between May and July 2020. Fig 1 details the flow diagram of the study participants. We excluded 86 patients on account of the study exclusion criteria. Finally, 375 patients were contacted for the phone interviews and 153 patients did not respond. The median timing for the follow-up phone calls after discharge was 122 days (IQR 109–158 days). Responses from the 222 patients were recorded and included in the present study.

### Baseline clinical characteristics of the cohort

A total of 222 patients discharged alive from the hospital post-acute COVID-19 infection responded to the follow-up phone survey. The demographic and clinical characteristics of these patients are summarized in Table 1. Men constituted 77% of the study cohort. The mean age of the cohort was 52.47 ± 13.95 years. A considerable proportion of the cohort was of Arab descent (61.3%) with the majority being non-smokers (89.2%), overweight or obese (71%). One-hundred and forty-one patients (63.5%) had pre-existing comorbidities with diabetes mellitus being the most common (47.7%), followed by hypertension (41%), cardiac disease (12.2%), lung disease (10.8%), dyslipidemia (5%) and kidney disease (3.6%). The majority of patients in our cohort endured moderate COVID-19 disease (46.4%), while the remaining had mild (12.2%), severe (21.6%), or critical disease (19.8%). Less than one-third of the patients were admitted to the ICU (30.2%). The mean length of stay in the hospital was 13.41 ± 11.27 days and that in the ICU was 3.15 ± 6.93 days.

**Table 1:** Characteristics of patients discharged post-COVID-19 (N = 222).

			**Frequency (n)**	**Percentage (%)**
**Gender**	Male		171	77.0
	Female		51	23.0
**Age (Mean±SD)**	52.47	(13.95)		
**Age group (years)**	18–34		19	8.6
	35–49		67	30.2
	50–66		100	45.0
	67+		36	16.2
**Nationality**	Saudi		87	39.2
	Non-Saudi		135	60.8
**Ethnicity**	Arab		136	61.3
	Indian		41	18.5
	Filipino		22	9.9
	Pakistani		16	7.2
	Other		6	2.7
**Smoking Status**	Smoker		3	1.4
	Non-Smoker		198	89.2
	Former Smoker		5	2.3
	Unknown		16	7.2
**BMI (*N = 209*)**	Underweight		2	1.0
	Normal		49	23.4
	Overweight		79	37.8
	Obese		79	37.8
**Pre-existing Comorbidities**	Yes		141	63.5
	No		81	36.5
**Number of Comorbidities *(N = 141)***	One		55	70.9
	Two		49	34.8
	Three		28	19.9
	Four		9	6.4
**Co-morbidities**	Diabetes		106	47.7
	Hypertension		91	41.0
	Dyslipidemia		11	5
	Cardiac disease		27	12.2
	Lung disease		24	10.8
	Renal disease		8	3.6
	Liver disease		2	0.9
	CVA		2	0.9
	Immunosuppression		1	0.5
**Admission type**	Ward		155	69.8
	ICU		67	30.2
**COVID19 Disease Severity**	Mild		27	12.2
	Moderate		103	46.4
	Severe		48	21.6
	Critical		44	19.8
**Chest X-ray abnormality**			210	94.6
**Mean ± SD**				
**Laboratory investigations**	ABS Neutrophils		7.56 ± 7.28	
	ABS Lymphocytes		1.11 ± 0.66	
	LDH (U/L)		700.17 ± 1183.93	
	Direct Bilirubin (μmol/L)		7.10 ± 21.76	
	Creatine Kinase (CK) (U/L)		471.66 ± 1324.43	
**Symptoms at follow-up**			125	56.3
**Duration of the majority for all**	1–7 days		66	32.0
**Symptoms *(N = 206)***				
	8–14 days		41	19.9
	15–21 days		33	16.0
	>21 days		66	32.0
**Return to Baseline (Pre-COVID)**	Yes		158	71.2
	No		64	28.8
**Hospital readmission**			16	7.2
**ER visit**			39	17.6
**Hospital admission (LOS)**			13.41 ± 11.27	
**ICU (LOS)**			3.15 ± 6.93	

### Prevalence of symptoms at follow-up

More than half of the cohort, 125 patients (56.3%) experienced unresolved symptoms after a month of discharge with 66 (29.7%) experiencing these new or persistent symptoms for >21 days (Table 1). Despite that, 71.2% of the cohort returned to their pre-COVID baseline state after a median of 4 months of follow-up. During this same period, 7.2% required hospital readmission and 17.6% re-visited the ER.

**Fig 2:**
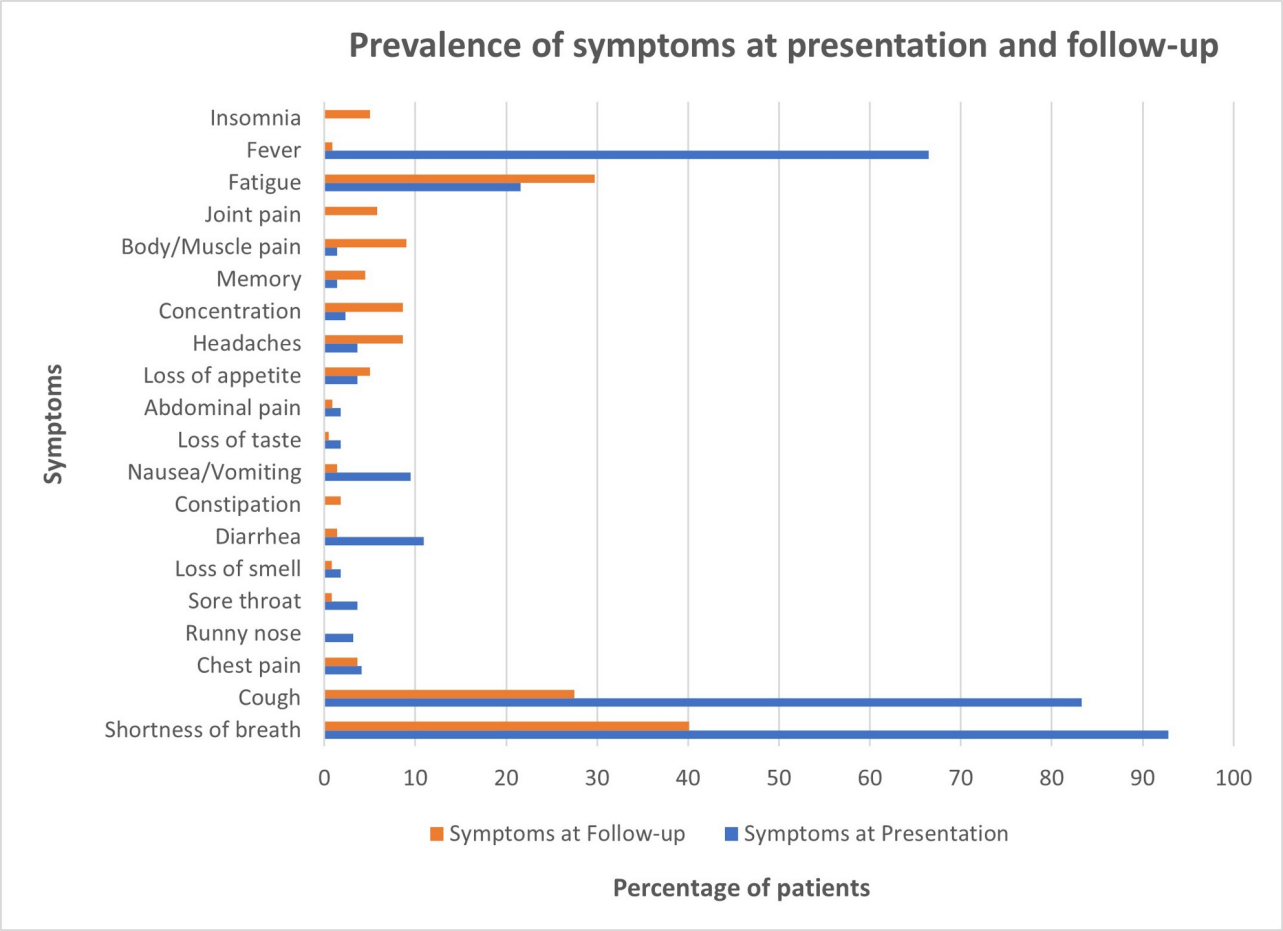
Prevalence of symptoms at presentation and follow-up (N = 125).

Approximately 40% of the patients reported the persistence of shortness of breath, followed by fatigue (30%) and cough (27.5%) (Fig 2 and S2 Table). Patients reported less frequently (<10%) of lasting muscle/body pain, difficulty concentrating, headaches, insomnia, joint pain, memory impairment, loss of appetite, chest pain, fever, abdominal pain, nausea/vomiting, constipation, diarrhea, loss of taste, loss of smell and sore throat.

Shortness of breath and cough were the two most prevalent symptoms at presentation as well as at follow-up. Among the neurological symptoms, headaches followed by difficulty concentrating were the most prevalent at presentation as well as at follow-up. In terms of evolution of symptoms, we observed that the prevalence of dyspnea, cough and fever decreased over the course of presentation to follow-up. However, other neurological and musculoskeletal symptoms, such as insomnia, fatigue, body/muscle pain, joint pain, memory impairment, concentration issues, headaches, and loss of appetite increased over time (Fig 2 and S2 Table).

### Predictors of persistent symptoms at follow-up

Because patients had different follow-up durations, a COX proportional hazards model was used to examine the risk factors associated with new or persistent symptoms at follow-up (Table 2). Female gender (adjusted Hazards ratio [aHR] 1.61; 95% CI 1.02–2.53; p = 0.04), liver disease (aHR 13.45; 95% CI 2.44–74.14; p = 0.003), hypertension (aHR 1.73; 95% CI 1.09–2.74; p = 0.02) and the length of hospital stay (aHR 1.04; 95% CI 1.01–1.07; p = 0.02) were found to be independently associated with new or persistent symptoms at follow-up. Interestingly, COVID-19 patients with pre-existing diabetes mellitus were less likely to experience lasting symptoms (aHR 0.54, 95% CI 0.34–0.87; p = 0.01). Patients who were given interferon β-1b based treatment had lower risk of reporting symptoms at follow-up compared to those who were not (aHR 0.28; 95% CI 0.14–0.57; p≤0.001).

**Table 2:** Multivariate Cox proportional hazards model of new or persistent symptoms at follow-up (N = 222).

		*B*	SE	*P value*	aHR (95% CI)
**Age**		-0.01	0.01	0.398	0.99 (0.98–1.01)
**Gender** (Female)		0.48	0.23	0.040	**1.61 (1.02–2.53)** *
**Co-morbidities**					
**Diabetes**		-0.61	0.24	0.011	**0.54 (0.34–0.87)** *
**Hypertension**		0.55	0.24	0.021	**1.73 (1.09–2.74)** *
**Lung disease**		0.21	0.31	0.493	1.23 (0.68–2.25)
**Liver disease**		2.60	0.87	0.003	**13.45 (2.44–74.14)** *
**ICU admission**		-0.03	0.77	0.966	0.97 (0.21–4.41)
**COVID Disease severity**	Mild Moderate Severe Critical	*Reference*			
		-0.33	0.44	0.461	0.72 (0.31–1.71)
		-0.37	0.52	0.473	0.69 (0.25–1.91)
		-0.20	0.62	0.751	0.82 (0.24–2.79)
**Treatments**					
**Triple Antiviral**		-1.27	0.36	≤0.001	**0.28 (0.14–0.57)** *
**Convalescent plasma**		0.37	0.41	0.372	1.45 (0.64–3.26)
**Tocilizumab**		0.52	0.30	0.076	1.69 (0.95–3.01)
**Seven category scale** ^#^	Scale 3				*Reference*
	Scale 4	0.47	0.46	0.310	1.59 (0.65–3.90)
	Scale 5–6	0.47	0.90	0.601	1.60 (0.27–9.38)
**Length of hospital stay (days)**		0.04	0.02	0.021	**1.04 (1.01–1.07)** *
**Length of ICU stay (days)**		-0.01	0.03	0.936	1.00 (0.95–1.05)

Event cases included in model = 125 and censored cases = 97, Time: Days since discharge; Model Fit: -2Log-Likelihood 1149.96 Chi-Square 51.157 (df = 17, p-value≤0.001)

^*^Significant at p<0.05

^#^Seven category scale: Scale 3: admitted to hospital not requiring supplemental oxygen, Scale 4: admitted to hospital requiring supplemental oxygen; Scale 5: admitted to hospital requiring HFNC or non-IMV or both; Scale 6: admitted to hospital requiring ECMO or IMV or both.

### Predictors of lack of return to baseline health state

Multivariable logistic regression analysis was used to identify risk factors associated with non-return to baseline pre-COVID-19 health state at a median of 4 months follow-up. The logistic regression was adjusted for age, gender, nationality, pre-existing morbidities, BMI, disease severity, severity category, treatment, ICU admission, hospital re-admission, ER visit, hospital stay (LOS) and ICU stay (LOS) (Table 3). Almost a third (31%) of patients falling in the age group of 50–66 years failed to return to their baseline pre-COVID-19 state (adjusted odds ratio [aOR] 10.05; 95% CI 1.33–76.06). Compared to non-Saudi individuals, the Saudi nationals were more likely to not return to their pre-COVID-19 health state (aOR 3.58; 95% CI 1.44–8.89). While 41.7% of patients with pre-existing lung disease did not return to their baseline state (aOR 4.93; 95% CI 1.33–18.27), those with diabetes mellitus were more likely to return to their pre-COVID-19 state (aOR 0.26, 95% CI 0.1–0.68). Notably, COVID-19 patients on interferon β-1b based treatment were 83% more likely to return to their baseline state after acute infection and only 20% of our patients on this treatment did not return back to normal (aOR 0.17; 95% CI 0.04–0.75). Our analysis also indicated that patients who visited the ER after discharge were at higher risk to not return to their pre-COVID-19 state (aOR 4.60; 95% CI 1.45–14.63).

**Table 3:** Predictors for non-return to baseline (pre-COVID-19) identified by multivariable logistic regression analysis (N = 64).

		n (%)	Crude OR (95% CI)	aOR (95% CI)^#^
**Gender**	Male ^a^	45/171 (26.3)	1	1
	Female	19/51 (37.1)	1.66 (0.86–3.22)	1.29 (0.50–3.35)
**Age**	18–34^a^	2/19 (10.5)	1	1
	35–49	21/67 (31.3)	3.88 (0.82–18.35)	7.02 (0.96–51.29)
	50–66	31/100 (31.0)	3.82 (0.83–17.55)	**10.05 (1.33–76.06)** *
	67+	10/36 (27.8)	3.27 (0.64–16.80)	5.05 (0.56–45.80)
**Nationality**	Non-Saudi ^a^	36/135 (26.7)	1	1
	Saudi	28/87 (32.2)	1.31 (0.72–2.35)	**3.58 (1.44–8.89)** *
**Pre-existing Comorbidities**				
**Dyslipidemia**		3/11 (27.3)	0.92 (0.24–3.59)	0.32 (0.05–2.23)
**Diabetes**		24/106 (23.1)	0.59 (0.32–1.06)	**0.26 (0.10–0.68)** *
**Hypertension**		27/91 (30.0)	1.10 (0.61–1.98)	1.69 (0.68–4.16)
**Cardiac disease**		8/27 (29.6)	1.05 (0.43–2.53)	1.28 (0.34–4.84)
**Renal disease**		1/8 (12.5)	0.34 (0.04–2.84)	0.41 (0.03–6.61)
**Lung disease**		10/24 (41.7)	1.91 (0.80–4.45)	**4.93 (1.33–18.27)** *
**Liver disease**		1/2 (50)	2.49 (0.15–40.46)	1.26 (0.03–57.53)
**BMI**	Normal ^a^	14/51 (27.5)	1	1
	Overweight	22/79 (27.8)	1.02 (0.46–2.24)	0.92 (0.35–2.44)
	Obese	24/79 (30.4)	1.15 (0.53–2.52)	0.85 (0.30–2.38)
**Disease severity**	Mild ^a^	5/27 (18.5)	1	1
	Moderate	25/103 (24.3)	1.41 (0.48–4.11)	1.19 (0.16–9.08)
	Severe	12/48 (25.0)	1.47 (0.46–4.73)	0.88 (0.08–9.80)
	Critical	22/44 (50.0)	**4.40 (1.41–13.71)** *	1.91 (0.12–30.30)
**Severity category Scale**	Scale 3 ^a^	4/23 (17.4)	1	1
	Scale 4	31/129 (24.0	1.50 (0.48–4.75)	0.58 (0.08–4.48)
	Scale 5–6	29/70 (41.4)	**3.36 (1.03–10.92)** *	7.44 (0.22–25.49)
**Treatments**	Triple Antiviral	6/30 (20.0)	0.58 (0.22–1.49)	**0.17 (0.04–0.75)** *
	Favipravir	19/50 (38.0)	1.73 (0.89–3.36)	2.46 (0.97–6.19)
	Plasma	5/12 (41.7)	1.83 (0.56–5.99)	1.04 (0.15–7.17)
	Tocilizumab	15/33 (45.5)	**2.38 (1.12–5.08)** *	1.42 (0.45–4.51)
	Steroids	55/182 (30.2)	1.49 (0.67–3.34)	1.02 (0.31–3.37)
**ICU admission**		29/67 (43.3)	**2.62 (1.42–4.83)** *	-
**Hospital re-admission**		9/16(56.3)	**3.53 (1.25–9.94)** *	1.16 (0.19–6.99)
**ER visit**		22/39 (56.4)	**4.31 (2.10–8.87)** *	**4.60 (1.45–14.63)** *
**Length of hospital stay**		18.25±15.71	**1.06 (1.03–1.09)** *	1.05 (0.98–1.11)
**Length of ICU stay**		5.47 ± 9.82	**1.07 (1.02–1.12)** *	0.99 (0.90–1.10)

*OR* Odds Ratio, *CI* Confidence Interval,

^*^Significant at p<0.005,

^*a*^ reference group,

^#^Logistic regression adjusted for age, gender, nationality, Pre-existing morbidities, BMI, disease severity, severity category, treatment, ICU admission, LOS, hospital re- admission, ER visit, ICU LOS. **Model Fit: *Hosmer and Lemeshow test (X***^***2***^ ***9*.*125; df = 8; P = 0*.*332); -2Log likelihood 185*.*63***

## Discussion

### Main findings

To our knowledge, this is the first report from the MENA region assessing a broad spectrum of functional outcomes in COVID-19 patients following hospital discharge. We observed that 56.3% of our cohort reported new or persistent symptoms at follow-up with most symptoms lasting over 21 days after diagnosis. At a median of 4 months follow-up after hospital discharge, shortness of breath, fatigue and cough were the main symptoms that prevailed in these patients. We also observed that more than one quarter of patients failed to return to their baseline health state after recovery from acute infection and 17.6% of the patients visited the ER after discharge. Female gender, pre-existing hypertension and longer hospital stay were associated with higher risk of new or persistent symptoms. Older age, pre-existing lung disease and post-discharge ER visits were predictors of delayed return to baseline health state. Interestingly, patients with diabetes and on triple antiviral therapy during hospital stay were less likely to experience new or persistent symptoms and more likely to return to their baseline health.

### Comparison to other studies

Although multiple recent studies have reported the long-term outcomes of COVID-19 survivors in the American [15], Asian [14] and European [4, 16–18] settings, very few have examined the predictors of PACS. In the largest post-acute COVID-19 study to date conducted in the Veteran Affairs hospitals in the US and involving 73,435 non-hospitalized COVID-19 survivors beyond the first 30 days of illness, people with COVID-19 were found to be at higher risk of death and health care resource utilization, and to exhibit a broad array of incident pulmonary and extrapulmonary clinical manifestations within 6 months after diagnosis [15]. In another cohort study from China, which included 1,733 discharged patients with COVID-19 with a median follow-up time after symptom onset of 186 (175–199) days, fatigue, or muscle weakness (63%) and sleep difficulties (26%) were the most commonly reported symptoms [14]. In this same cohort, 76% of patients reported at least one symptom at 6 months after symptom onset, and the proportion was higher in women. After multivariable adjustment, women had a higher risk for anxiety or depression [OR 1·80 (1·39–2·34)], and for fatigue or muscle weakness [OR 1·33 (1·05–1·67)] compared with men. Severity of COVID-19 was also associated with persistent symptoms with OR 2·42 (1·15 to 5·08). In another Italian study, 87.4% of the cohort experienced the persistence of at least one symptom after recovery at their 2-month follow-up [4].

A systematic review and meta-analysis of studies assessing long-term effects of COVID-19 identified fatigue (58%), headache (44%), attention disorder (27%), hair loss (25%), and dyspnea (24%) as the five most common persistent symptoms [19]. However, these meta-analyses showed medium-to-high heterogeneity as the included studies had not stratified patients by gender, age, previous comorbidities, severity of COVID-19 (ranging from asymptomatic to severe), and duration of each symptom, which however was accomplished in our study. The varying prevalence of the various symptoms across these studies may be attributed to the difference in patient selection criteria and data collection methods.

A recent report analyzing data from over 4000 COVID-19 survivors using the COVID Symptom Study app identified female gender, increasing age, body mass index, and patients who experienced more than five symptoms during the first week of infection at risk of long COVID [20]. Like our study, other previous investigations identified female gender as an independent predictor of unresolved symptoms [14, 21]. Previous studies have also outlined an association between female gender and long COVID [14, 20], post-exertional polypnea [22], persisting fatigue [14, 22, 23], anxiety or depression [14, 24] and decreased rates of recovery [24]. However, there is no clear pathophysiology why females are more susceptible to prolonged effects of the disease than males. Viral-induced autoimmunity is a potential immunopathological mechanism underlying PACS and the higher representation of women in autoimmune diseases [25] may explain the gender differences in immunological response between acute COVID-19 and post-COVID-19 syndrome.

In addition, patients in the age group of 50–66 years, Saudi nationals, those with pre-existing lung disease and those who visited the ER after discharge were less likely to return to their baseline health state. Similarly, in the large cohort from China, age was positively associated with fatigue and muscle weakness (OR 1·17; 1·07–1·27) per 10-year increase of age [14]. Patients who require ER visits after hospitalization were associated with poor long-term effects and this could be related to the effect of pandemic on rehabilitation program and superimposed infection.

Surprisingly, COVID-19 patients with pre-existing diabetes mellitus were less likely to experience lasting symptoms and were more likely to return to their baseline health state. This may be a chance finding or due to selection bias given that patients with diabetes experience severe disease and are more likely to stay longer in the ICU or die. Patients with diabetes who survived and participated in this survey could therefore be a selected sample with controlled diabetes and exhibit the healthy user effect. The observation that triple antiviral therapy was associated with lower risk of PACS may be explained by the effect of this therapy on the viremia level in the acute phase of the disease resulting in less inflammatory consequences at follow-up. Type I interferon (IFN) signaling is crucial particularly in the early phase of viral infection for optimal virus control [26] and IFNβ may partially be responsible for this early antiviral response [27]. On the other hand, a loss of function gene variant governing type I IFN immunity predisposes patients to life-threatening COVID-19 pneumonia [28]. Among types I and II recombinant interferons (including IFN β-1a, IFN β-1b, pegylated IFN α-2a and IFN γ-1b), interferon-β1b (Betaferon) was found to demonstrate the most potent anti-SARS-CoV-2 activity [29]. Since the viral load is already high at symptom onset [30], antiviral therapy may be initiated early in COVID-19 patients.

### Strengths and limitations

Our study has multiple strengths. To our knowledge, it is the first report from the MENA region to describe long-term symptoms experienced by COVID-19 survivors after hospitalization and one of very few studies that examined predictors of PACS. Our patients were subjected to a unified COVID-19 treatment protocol which reduced the variations in the clinical management of patients allowing the comparison of clinical outcomes in a uniform cohort. Moreover, we addressed multiple limitations that affected previous studies such as lack of baseline assessment or use of diagnostic codes. Our study also has certain limitations. Firstly, the sample size of our cohort is relatively smaller and is a single center experience. Although we did not observe an association between disease severity and PACS, our analysis could be underpowered. Secondly, the exclusion of non-hospitalized patients affects the generalizability of our findings to patients with mild COVID-19. The risk of PACS in non-hospitalized patients is typically low [31].

## Conclusions

At a median of 4 months after hospital discharge, shortness of breath, fatigue and cough were the main symptoms experienced by patients who had recovered from COVID-19. Nearly 30% of these patients did not return to their baseline pre-COVID-19 state and 17.6% had to visit the ER for this reason. We identified multiple risk factors for new or persistent symptoms and lack of return to baseline health state after acute COVID-19 infection. Longer follow-up studies in a larger population are warranted to understand the persistent chronic symptoms experienced by these long-haulers to develop guidelines for their improved clinical management and care.

## Supporting information

S1 Questionnaire Arabic questionnaire. (DOCX)

S2 Questionnaire English questionnaire. (DOCX)

S1 Table Patient characteristics by follow-up (N = 375). *Significant at p<0.05 using Chi-square and T-tests.
(DOCX)

S2 Table Prevalence of symptoms at presentation and follow-up (N = 222). (DOCX)

S3 Table Comparison of patient admission characteristics with symptoms at follow-up*. Using Chi-Square and Fisher’s exact test analysis for categorical variables and T-test and Mann-Whitney U for continuous variables #Seven category scale: Scale 3: admitted to hospital not requiring supplemental oxygen, Scale 4: admitted to hospital requiring supplemental oxygen; Scale 5: admitted to hospital requiring HFNC or non-IMV or both; Scale 6: admitted to hospital requiring ECMO or IMV or both.
(DOCX)

S4 Table Comparison of treatment modality and symptoms at follow-up. * Using Chi-Square analysis and Fisher’s exact test where appropriate # Seven category scale: Scale 3: admitted to hospital not requiring supplemental oxygen, Scale 4: admitted to hospital requiring supplemental oxygen; Scale 5: admitted to hospital requiring HFNC or non-IMV or both; Scale 6: admitted to hospital requiring ECMO or IMV or both.
(DOCX)
