# Supplementary material for: Prevalence and predictors of Post-Acute COVID-19 Syndrome (PACS) after hospital discharge: A cohort study with 4 months median follow-up
Source: PLoS One. 2021 Dec 7;16(12):e0260568. doi: 10.1371/journal.pone.0260568 (PMC8651136; doi:10.1371/journal.pone.0260568)
Supplement: S1 Questionnaire — (DOCX) [file pone.0260568.s001.docx]

**Arabic Questionnaire**

السلام عليكم ورحمة الله وبركاته :

أنا الطبيب (...) من مدينة الملك فهد الطبية، قسم أمراض الباطنة، نجري بحثا حول تجارب مرضى فايروس كورونا COVID-19 قصيرة وبعيدة المدى بعد خروجهم من المستشفى ونود مشاركتك . سيتم استخدام المعلومات لوصف الأعراض التي استمرت بعد الخروج من المستشفى ومدة بقائها ومقارنتها بشدة الالتهاب الرئوي، المضاعفات المصاحبة والرعاية الطبية التي تمت خلال التنويم وقياس مدى العلاقة بينها وبين الأمراض المزمنة المصاحبة إن وجدت .

لا يتوجب عليك الإجابة على جميع الأسئلة ولك الحق في إنهاء المقابلة الهاتفية في أي وقت. ستكون مدة المقابلة ١٠ دقائق وجميع المعلومات ستكون بسرية تامة. وفي حال وجود أي سؤال عن البحث أو الاستبانة يمكنك التواصل مع مركز الأبحاث بمدينة الملك فهد الطبية عن طريق الرقم الآتي :

1. السجل الطبي للمريض خلال المقابلة الهاتفية بعد الخروج من المستشفى:

| **تاريخ المقابلة الهاتفية** |  |
| --- | --- |
| **الاستبانات**  **المصدقة ( المعتمدة ) المستخدمة في البحث**  **Validated Questionnaires** | 1. Fatigability  التعب/ الإجهاد   Chronic Fatigability syndrome (CFS) questionnaire  Timbol CR, Baraniuk JN. Chronic fatigue syndrome in the emergency department. *Open Access Emerg Med*. 2019;11:15-28  <https://doi.org/10.2147/OAEM.S176843>  <https://www.dovepress.com/chronic-fatigue-syndrome-in-the-emergency-department-peer-reviewed-fulltext-article-OAEM>.   1. Exercise Tolerance مقياس احتمال التمارين   Metabolic equivalent of task (MET) score  Böhmer AB, Wappler F, Zwissler B. Preoperative risk assessment--from routine tests to individualized investigation. *Dtsch Arztebl Int*. 2014;111(25):437-446. doi:10.3238/arztebl.2014.0437  <https://pubmed.ncbi.nlm.nih.gov/25008311/>  <https://onlinelibrary.wiley.com/doi/epdf/10.1002/clc.4960130809>   1. Exertional Dyspneaضيق التنفس عند الإجهاد   Medical research council (MRC) score: For exertional dyspnea  Luciana Paladini, Rick Hodder, Isabella Cecchini, Vincenzo Bellia, Raffaele Antonelli Incalzi,  The MRC dyspnoea scale by telephone interview to monitor health status in elderly COPD patients,  Respiratory Medicine,  Volume 104, Issue 7, 2010, Pages 1027-1034, ISSN 0954-6111,  https://doi.org/10.1016/j.rmed.2009.12.012.  <https://www.sciencedirect.com/science/article/pii/S0954611110000028>   1. Mental health الصحة النفسية   Who five well being index (WHO-5)  Topp CW, Østergaard SD, Søndergaard S, Bech P. The WHO-5 Well-Being Index: a systematic review of the literature. *Psychother Psychosom*. 2015;84(3):167-176. doi:10.1159/000376585  <https://www.karger.com/Article/Pdf/376585> |
| **الأعراض عند المتابعة ( الأعراض التي لم تزول )** | \| **أعراض الجهاز الهضمي** \| \| **الأعراض القلبية والتنفسية** \| \| \| --- \| --- \| --- \| --- \| \|  \| إسهال \|  \| ضيق في التنفس \| \|  \| الغثيان / التقيؤ \|  \| ضيق في التنفس مع المجهود \| \|  \| فقدان حاسة التذوق \|  \| ألم في الصدر \| \|  \| ألم في البطن \|  \| السعال \| \|  \| فقدان الشهية \|  \| احتقان أو سيلان الأنف \| \|  \| إمساك \|  \| التهاب الحلق \| \| **أعراض الجهاز العصبي** \| \|  \| فقدان حاسة الشم \| \|  \| صداع \|  \| انسداد الأذن \| \|  \| فقدان التركيز \| **أعراض الجهاز العضلي الهيكلي** \| \| \|  \| اختلال بالذاكرة \|  \| ألم عضلي \| \|  \| دوخة / دوار \|  \| ألم في المفاصل \| \|  \| اختلال في النظر / تغيم بالرؤية \| \| **أعراض أخرى** \| \| \|  \| التعب / الإجهاد \| \|  \| ارتفاع حرارة الجسم \| \|  \| التعرق الليلي \| \|  \| طفح جلدي \| \|  \| الأرق \| |
| **زوال الأعراض ( العودة للحياة الطبيعية قبل الإصابة بفايروس كورونا )** | - نعم - لا |
| **المدة الزمنية لزوال الأعراض ( بالأيام )** | - 1-7 - 8-14 - 14-21 - > 21 |

**الاستبانات المصدقة ( المعتمدة ) المستخدمة في البحث**

**( Validated Questionnaires )**

1. ضيق التنفس عند الإجهاد MRC questionnaires

| مقياسMRC لضيق التنفس | | |
| --- | --- | --- |
| MRC تصنيف | الوصف | مقياس الشدة |
| 1 | ضيق في التنفس مع بذل المجهود العالي كالتمارين | خفيف |
| 2 | ضيق في التنفس عند زيادة سرعة المشي أو طلوع مرتفع | معتدل |
| 3 | المشي بسرعة أبطئ من سرعة مشي من هم بنفس العمر أو التوقف لالتقاط النفس عند المشي |  |
| 4 | التوقف لالتقاط النفس عند المشي لمسافة ١٠٠ متر | شديد |
| 5 | ضيق في التنفس يمنعه من الخروج م المنزل أو حتى عند ارتداء الملابس |  |

1. الصحة النفسية : WHO-5 questionnaires

| **خلال آخر أسبوعين** | كل الوقت | معظم الوقت | أكثر من نصف الوقت | أقل من نصف الوقت | أحيانا | نادرا |
| --- | --- | --- | --- | --- | --- | --- |
| أشعر بالبهجة ومعنوياتي عالية | 5 | 4 | 3 | 2 | 1 | 0 |
| أشعر بالهدوء والاسترخاء | 5 | 4 | 3 | 2 | 1 | 0 |
| أشعر بالنشاط والقوة | 5 | 4 | 3 | 2 | 1 | 0 |
| أستيقظ من النوم وأشعر بالانتعاش والراحة | 5 | 4 | 3 | 2 | 1 | 0 |
| حياتي اليومية مليئة بالأشياء المهمة | 5 | 4 | 3 | 2 | 1 | 0 |

|  | المجموع الكلي |
| --- | --- |

1. التعب / الإجهاد :

Chronic Fatigability syndrome (CFS) questionnaire

| مستوى الشدة خلال الأشهر الماضية | | | | | أكثر من نصف الوقت | | الأعراض |
| --- | --- | --- | --- | --- | --- | --- | --- |
| شديد | معتدل | خفيف | طفيف | لا يوجد | لا | نعم |  |
| 4 | 3 | 2 | 1 | 0 | لا | نعم | الخمول |
| 4 | 3 | 2 | 1 | 0 | لا | نعم | اختلال بالذاكرة أو التركيز خلال فترة وجيزة |
| 4 | 3 | 2 | 1 | 0 | لا | نعم | التهاب الحلق |
| 4 | 3 | 2 | 1 | 0 | لا | نعم | التهاب الغدد اللمفاوية ( العنق ، الإبطين ، الفخذ ) |
| 4 | 3 | 2 | 1 | 0 | لا | نعم | ألم في العضلات |
| 4 | 3 | 2 | 1 | 0 | لا | نعم | ألم في المفاصل |
| 4 | 3 | 2 | 1 | 0 | لا | نعم | صداع |
| 4 | 3 | 2 | 1 | 0 | لا | نعم | صعوبة في النوم أو نوم غير منعش |
| 4 | 3 | 2 | 1 | 0 | لا | نعم | الإجهاد الشديد بعد ممارسة التمارين أو مجهود بسيط |

| المجموع |
| --- |
|  |
| المجموع الكلي : |

|  | | التعب / الإجهاد | |
| --- | --- | --- | --- |
|  |  | لا يوجد ، طفيف ، خفيف | معتدل ، شديد |
| المجموع الكلي | 0-13 | طبيعي | التعب المزمن مجهول السبب |
|  | 14-32 | شبيهة متلازمة التعب المزمن | متلازمة التعب المزمن |

1. مقياس احتمال التمارين :

Metabolic equivalents (MET) of various activities

| Metabolic equivalents (MET) of various activities  مكافئات التمثيل الغذائي باختلاف الأنشطة | |
| --- | --- |
| MET | نوع النشاط |
| 1 | القراءة ، مشاهدة التلفاز |
|  | الأكل ، ارتداء الملابس |
| 2-3 | المشي على أرض مسطحة لمسافة ٣-٤ كلم/ساعة |
|  | الأعمال المنزلية الخفيفة |
| 4 | تسلق / طلوع بضع درجات |
|  | لمشي على أرض مسطحة لمسافة ٦ كلم/ساعة |
|  | الركض لمسافة قصيرة |
|  | الأعمال المنزلية الثقيلة |
|  | الرياضات المتوسطة الشدة ( الجولف ، الرقص ) |
| >10 | الرياضات عالية الشدة ( كرق القدم ، التنس ) |
| >10 ممتاز, 9-4 في المتوسط , 3-2 أقل من المتوسط , < 2 ضعيف | |
