# Supplementary material for: Prevalence and predictors of Post-Acute COVID-19 Syndrome (PACS) after hospital discharge: A cohort study with 4 months median follow-up
Source: PLoS One. 2021 Dec 7;16(12):e0260568. doi: 10.1371/journal.pone.0260568 (PMC8651136; doi:10.1371/journal.pone.0260568)
Supplement: S2 Questionnaire — (DOCX) [file pone.0260568.s002.docx]

**English Questionnaire**

- Date of interview :
- Return to baseline:
- Yes
- No
- Duration for symptoms resolution : in days
- 1-7
- 8-14
- 14-21
- > 21
- Symptoms at Follow up :
- Respiratory :
- GI :
- MSK :
- Neuro :
- Miscellaneous :
- MRC :
- MRC Grade , severity :

| MRC Dyspnoea scale by Telephone Interview | | |
| --- | --- | --- |
| MRC Grade | Description in | MRC severity grouping |
| 1 | Breathlessness with strenuous exercise | Mild |
| 2 | Short of breath when hurrying on the level or walking a slight hill. | Moderate |
| 3 | Walks slower than people of the same age on the level , or stops for breath while walking at own pace on the level. |  |
| 4 | Stops for a breath after walking a 100m. | Severe |
| 5 | Too breathless to leave the house or breathless when dressing. |  |

- WHO-5
- Total final ( multiply by 4 ) :

| The WHO-5 Questionnaire | | | | | | |
| --- | --- | --- | --- | --- | --- | --- |
| Over the past 2 weeks | All of the time | Most of the time | More than half of the time | Less than half the time | Some of the times | At no time |
| 1  ... I have felt cheerful and in good spirits | 5 | 4 | 3 | 2 | 1 | 0 |
| 2  ... I have felt calm and relaxed | 5 | 4 | 3 | 2 | 1 | 0 |
| 3  ... I have felt active and vigorous | 5 | 4 | 3 | 2 | 1 | 0 |
| 4  ... I woke up feeling fresh and rested | 5 | 4 | 3 | 2 | 1 | 0 |
| 5  ... my daily life has been filled with things that interest me | 5 | 4 | 3 | 2 | 1 | 0 |
| Scoring principle: The raw score ranging from 0 to 25 is multiplied by 4 to give the final score from 0 representing the worst imaginable well-being to 100 representing the best imaginable well-being. | | | | | | |

- CFS
- Total by Number :
- Total by name :

| Chronic Fatigability syndrome CFS | | | | | | | | |
| --- | --- | --- | --- | --- | --- | --- | --- | --- |
| Symptom | Is this a problem more than half of the time ? | | Severity in the past few Months | | | | | score |
|  | Yes | No | None  0 | Trival  1 | Mild  2 | Moderate  3 | Severe  4 |  |
| Fatigue | Yes | No | 0 | 1 | 2 | 3 | 4 |  |
| Short term problems with memory or concentrating | Yes | No | 0 | 1 | 2 | 3 | 4 | Sum |
| Sore throat | Yes | No | 0 | 1 | 2 | 3 | 4 |  |
| Sore lymph nodes  ( neck,armpits,groin) | Yes | No | 0 | 1 | 2 | 3 | 4 |  |
| Muscle pain | Yes | No | 0 | 1 | 2 | 3 | 4 |  |
| Joint pain | Yes | No | 0 | 1 | 2 | 3 | 4 |  |
| Headache | Yes | No | 0 | 1 | 2 | 3 | 4 |  |
| Difficulty sleeping or unrefreshing sleep | Yes | No | 0 | 1 | 2 | 3 | 4 |  |
| Extreme fatigue after exercise or mild exertion | Yes | No | 0 | 1 | 2 | 3 | 4 |  |

|  | | Fatigue | |
| --- | --- | --- | --- |
|  |  | None, Trivial, Mild | Moderate, Severe |
| Sum of ancillary criteria (Sum8) | 0-13 | Normal | Chronic idiopathic fatigue |
|  | 14-32 | CFS-like with insufficient fatigue syndrome. | Chronic fatigue syndrome |

- MET
- Total by class :
- > 10 , Excellent
- 4-9 , Average
- 2-3 . below average
- < 2 , Poor

| Metabolic equivalents (MET) of various activities | |
| --- | --- |
| MET | Activity |
| 1 | Reading, watching television |
|  | Eating, getting dressed |
| 2-3 | Walking on level ground at 3-4 km/h |
|  | Light housework |
| 4 | Climbing a few stairs |
|  | Walking on level ground at ca. 6 km/h |
|  | Running (Short distances) |
|  | Heavy household chores |
|  | Moderately strenuous sports (Golf, dancing) |
| >10 | Highly strenuous sports (Tennis, soccer) |
| >10 Excellent, 9-4 Average, 3-2 below average, < 2 poor | |
