# Supplementary material for: Prevalence and predictors of Post-Acute COVID-19 Syndrome (PACS) after hospital discharge: A cohort study with 4 months median follow-up
Source: PLoS One. 2021 Dec 7;16(12):e0260568. doi: 10.1371/journal.pone.0260568 (PMC8651136; doi:10.1371/journal.pone.0260568)
Supplement: S1 Table — *Significant at p<0.05 using Chi-square and T-tests. (DOCX) [file pone.0260568.s003.docx]

**S1 Table: Patient Characteristics by Follow-up (N=375)**

|  | | **Follow-up n (%)** | **No Follow-up n (%)** | ***P-*Value*** |
| --- | --- | --- | --- | --- |
| **Gender** | Male | 171 (59.0) | 119 (41.0) | 0.864 |
|  | Female | 51 (60.0) | 34 (40.0) |  |
| **Nationality** | Saudi | 87 (60.4) | 57 (39.6) | 0.705 |
|  | Non-Saudi | 135 (58.4) | 96 (41.6) |  |
| **Age (Mean ± SD)** | | 52.47 (13.95) | 52.30 (14.06) | 0.909 |
| **BMI (Mean ± SD)** | | 29.49 (6.10) | 29.58 (5.79) | 0.895 |
| **Co-morbidities (Yes)** | Yes | 141 (59.2) | 97 (40.8) | 0.982 |
|  | No | 81 (59.1) | 56 (40.9) |  |
| **Disease Severity** | Mild | 27 (58.7) | 19 (41.3) | 0.165 |
|  | Moderate | 103 (62.0) | 63 (38.0) |  |
|  | Severe | 48 (50.0) | 48 (50.0) |  |
|  | Critical | 44 (65.7) | 23 (34.3) |  |
| **Admission type** | Ward | 155 (57.4) | 115 (42.6) | 0.257 |
|  | ICU | 67 (63.8) | 38 (36.2) |  |
| **Length of stay (Mean ± SD)** | | 13.41 (11.27) | 13.35 (8.90) | 0.962 |

*Significant at p<0.05 using Chi-square and T-tests.
