# Supplementary material for: Prevalence and predictors of Post-Acute COVID-19 Syndrome (PACS) after hospital discharge: A cohort study with 4 months median follow-up
Source: PLoS One. 2021 Dec 7;16(12):e0260568. doi: 10.1371/journal.pone.0260568 (PMC8651136; doi:10.1371/journal.pone.0260568)
Supplement: S2 Table — (DOCX) [file pone.0260568.s004.docx]

**S2 Table: Prevalence of symptoms at presentation and follow-up (N=222)**

| **Symptom** | **Presentation n (%)** | **Follow-up n (%)** |
| --- | --- | --- |
| **Respiratory** |  |  |
| Shortness of breath | 205 (92.8) | 89 (40.1) |
| Cough | 184 (83.3) | 61 (27.5) |
| Chest pain | 9 (4.1) | 8 (3.6) |
| Runny nose | 7 (3.2) | 0 |
| Sore throat | 8 (3.6) | 1 (0.5) |
| Loss of smell | 4 (1.8) | 1 (0.5) |
| Clogged ears | 1 (0.5) | 1 (0.5) |
| **Gastroenterology** |  |  |
| Diarrhea | 24 (10.9) | 3 (1.4) |
| Constipation | 0 | 4 (1.8) |
| Nausea/Vomiting | 21 (9.5) | 3 (1.4) |
| Loss of taste | 4 (1.8) | 1 (0.5) |
| Abdominal pain | 4 (1.8) | 2 (0.9) |
| Loss of appetite | 8 (3.6) | 11 (5) |
| **Neurological** |  |  |
| Headaches | 8 (3.6) | 19 (8.6) |
| Concentration | 5 (2.3) | 19 (8.6) |
| Dizziness | 3 (1.4) | 2 (0.9) |
| Memory | 3 (1.4) | 10 (4.5) |
| Blurry vision | 0 | 1 (0.5) |
| **Musculoskeletal** |  |  |
| Body/Muscle pain | 3 (1.4) | 20 (9) |
| Joint pain | 0 | 12 (5.8) |
| **Miscellaneous** |  |  |
| Fatigue | 48 (21.6) | 66 (29.7) |
| Fever | 147 (66.5) | 2 (0.9) |
| Night sweats | 4 (1.8) | 0 |
| Rash | 0 | 1 (0.5) |
| Insomnia | 0 | 11 (5) |
