# Supplementary material for: Prevalence and predictors of Post-Acute COVID-19 Syndrome (PACS) after hospital discharge: A cohort study with 4 months median follow-up
Source: PLoS One. 2021 Dec 7;16(12):e0260568. doi: 10.1371/journal.pone.0260568 (PMC8651136; doi:10.1371/journal.pone.0260568)
Supplement: S3 Table — Using Chi-Square and Fisher’s exact test analysis for categorical variables and T-test and Mann-Whitney U for continuous variables #Seven category scale: Scale 3: admitted to hospital not requiring supplemental oxygen, Scale 4: admitted to hospital requiring supplemental oxygen; Scale 5: admitted to hospital requiring HFNC or non-IMV or both; Scale 6: admitted to hospital requiring ECMO or IMV or both. (DOCX) [file pone.0260568.s005.docx]

**S3 Table: Comparison of patient admission characteristics with symptoms at follow-up**

|  | | **Symptoms at follow up,**  **n (%)** | | **Chi-Square χ^2^** | ***P-*Value^*^** |
| --- | --- | --- | --- | --- | --- |
|  | | **Yes** | **No** |  |  |
| **Gender** | Male | 91 (53.2) | 80 (46.8) | 4.897 | **0.039** |
|  | Female | 34 (66.7) | 17 (33.3) |  |  |
| **Age (years)** | 18-34  35-49  50-66  67+ | 11 (57.9)  43 (64.2)  51 (51.0)  20 (55.6) | 8 (42.1)  24 (35.8)  49 (49)  16 (44.4) | 2.860 | 0.414 |
| **Nationality** | Saudi | 49 (56.3) | 38 (43.7) | 0.021 | 0.885 |
|  | Non-Saudi | 76 (56.3) | 59 (43.7) |  |  |
| **Ethnicity** | Arab | 76 (55.9) | 60 (44.1) | 0.678 | 0.954 |
|  | Indian | 24 (58.5) | 17 (41.5) |  |  |
|  | Filipino | 13 (59.1) | 9 (40.9) |  |  |
|  | Pakistani | 8 (50.0) | 8 (50.0) |  |  |
|  | European | 2 (33.3) | 4 (66.7) |  |  |
| **Smoking Status** | Smoker | 2 (66.7) | 1 (33.3) | 3.198 | 0.362 |
|  | Non-Smoker | 114 (57.6) | 80 (42.4) |  |  |
|  | Former Smoker | 1 (20.0) | 4 (80.0) |  |  |
|  | Unknown | 8 (50.0) | 8 (80.0) |  |  |
| **BMI** | Underweight | 2 (100) | 0 | 2.516 | 0.472 |
|  | Normal | 28 (57.1) | 21 (42.9) |  |  |
|  | Overweight | 47 (59.5) | 32 (40.5) |  |  |
|  | Obese | 41 (51.9) | 38 (48.1) |  |  |
| **Existing Co-morbidity** |  | 72 (51.1) | 69 (48.9) | 4.317 | **0.038** |
|  | Diabetes | 49 (47.1) | 55 (52.9) | 6.718 | **<0.010** |
|  | Hypertension | 47 (52.2) | 43 (47.8) | 1.026 | 0.311 |
|  | Dyslipidemia | 5 (45.5) | 6 (54.5) | 0.554 | 0.457 |
|  | Cardiac disease | 14 (51.9) | 13 (48.1) | 0.248 | 0.619 |
|  | Lung disease | 16 (66.7) | 8 (33.3) | 1.174 | 0.279 |
|  | Renal disease | 4 (50.0) | 4 (50.0) | 0.134 | 0.714 |
|  | Liver disease | 2 (100) | 0 | 1.566 | 0.211 |
|  | CVA | 2 (100) | 0 | 1.566 | 0.211 |
| **Admission type** | Ward | 80 (51.6) | 75 (48.4) | 4.598 | **0.032** |
|  | ICU | 45 (67.2) | 22 (32.8) |  |  |
| **COVID19 Disease Severity** | Mild | 11 (40.7) | 16 (59.3) | 7.387 | 0.061 |
|  | Moderate | 53 (51.5) | 50 (48.5) |  |  |
|  | Severe | 32 (66.7) | 16 (33.3) |  |  |
|  | Critical | 29 (65.9) | 15 (34.1) |  |  |
| **Chest x-ray abnormality** | Yes | 110 (56.1) | 86 (43.9) | 2.371 | 0.124 |
|  | No | 4 (33.3) | 8 (66.7) |  |  |
| **Seven category scale^#^** | Scale 3  Scale 4  Scale 5-6 | 9 (39.1)  69 (53.5)  47 (67.1) | 14 (60.9)  60 (46.5)  23 (32.9) | 6.516 | **0.038** |
| **ER visit** | Yes  No | 32 (82.1)  93 (51.1) | 7 (17.9)  89 (48.9) | 12.524 | **<0.001** |
|  |  | **Symptoms at follow up** | | **Mann-Whitney *U*** |  |
|  |  | **Yes** | **No** |  |  |
|  | Mean ±SD | N (Mean Rank) | N (Mean Rank) |  |  |
| **Length of stay** | 13.41 ± 11.27 | 114 (115.42) | 94 (91.26) | 4113.50 | **0.004** |
| **ICU Length of stay** | 3.15 ± 6.93 | 125 (117.86) | 97 (103.31) | 5268.00 | **0.039** |

*^*^ ^Using Chi-Square and Fisher’s exact test analysis for categorical variables and T-test and Mann-Whitney U for continuous variables^* *^#Seven category scale: Scale 3: admitted to hospital not requiring supplemental oxygen, Scale 4: admitted to hospital requiring supplemental oxygen; Scale 5: admitted to hospital requiring HFNC or non-IMV or both; Scale 6: admitted to hospital requiring ECMO or IMV or both^*
