# Supplementary material for: Prevalence and predictors of Post-Acute COVID-19 Syndrome (PACS) after hospital discharge: A cohort study with 4 months median follow-up
Source: PLoS One. 2021 Dec 7;16(12):e0260568. doi: 10.1371/journal.pone.0260568 (PMC8651136; doi:10.1371/journal.pone.0260568)
Supplement: S4 Table — * Using Chi-Square analysis and Fisher’s exact test where appropriate # Seven category scale: Scale 3: admitted to hospital not requiring supplemental oxygen, Scale 4: admitted to hospital requiring supplemental oxygen; Scale 5: admitted to hospital requiring HFNC or non-IMV or both; Scale 6: admitted to hospital requiring ECMO or IMV or both. (DOCX) [file pone.0260568.s006.docx]

**S4 Table: Comparison of treatment modality and symptoms at follow-up**

| **Treatment** |  | **Frequency**  **n (%)** | **Symptoms at follow-up** | | ***Chi-Square χ^2^*** | ***P*-Value^*^** |
| --- | --- | --- | --- | --- | --- | --- |
|  |  |  | **Yes** | **No** |  |  |
| **Triple antiviral** |  | 30 (13.5) | 9 (30.0) | 21 (70.0) | 9.757 | **0.002** |
| **Favipravir** |  | 50 (22.5) | 33 (66.0) | 17 (34.0) | 2.465 | 0.116 |
| **Convalescent Plasma** | | 12 (5.4) | 9 (75.0) | 3 (25.0) | 1.802 | 0.179 |
| **Tocilizumab** | | 33 (14.9) | 24 (72.7) | 9 (27.3) | 4.248 | **0.039** |
| **Steroids** | | 182 (82) | 105 (57.7) | 77 (42.3) | 0.789 | 0.374 |
| **Oxygen Therapy** | None | 23 (10.3) | 9 (39.1) | 14 (60.9) |  |  |
|  | <15 L/min O_2_ | 129 (58.1) | 69 (53.5) | 60 (46.5) | 7.290 | 0.063 |
|  | HFNC | 31 (14.0) | 19 (61.3) | 12 (38.7) |  |  |
|  | Intubation/MV | 39 (17.6) | 28 (71.8) | 11 (28.2) |  |  |
| **Seven Category Scale^#^** | Scale 3  Scale 4  Scale 5 | 32 (10.4) | 9 (39.1 | 14 (60.9) | 6.516 | 0.038 |
|  |  | 129 (58.1) | 69 (53.5) | 60 (46.5) |  |  |
|  |  | 70 (31.5) | 47 (67.1) | 23 (32.9) |  |  |

*^*^ ^Using Chi-Square analysis and Fisher’s exact test where appropriate # Seven category scale: Scale 3: admitted to hospital not requiring supplemental oxygen, Scale 4: admitted to hospital requiring supplemental oxygen; Scale 5: admitted to hospital requiring HFNC or non-IMV or both; Scale 6: admitted to hospital requiring ECMO or IMV or both^*
